# Parliament and the Profession

**Published:** 1916-04-08

**Authors:** 


### PARLIAMENT AND THE PROFESSION.

### Conscientious Objectors as Hospital Orderlies.

Mr. Tennant emphatically stated that it was not to be the policy of the Government to employ conscientious objectors as orderlies at military hospitals. The matter arose out of a question asked by Mr. Newdegate which suggested that 200 orderlies had been transferred from a military hospital to undertake duties elsewhere, and 200 conscientious objectors of military age had been appointed to take their place. Mr. Tennant denied knowledge of this procedure.

### Reduction in Medical Fees.

The main object of most of the fourteen clauses of Part II. of the Local Government (Emergency Provisions) Bill is to promote a variety of small economies in Local Government administration which will release a great deal of labour, and in the aggregate effect the saving of a considerable sum of money. Clause 5 embodies the recommendation made by the Committee on Retrenchment in Public Expenditure that the fee paid to a medical practitioner for the notification of infectious diseases should be reduced from 2s. 6d. to 1s., and the Bill passed its second reading on March 29 without any objection being made to this proposal. In moving the second reading, Mr. Hayes Fisher said that one of the most fertile fields in which economy could be exercised was the field of Poor-Law accounts and returns. As a result of a conference between representatives of Boards of Guardians and the Metropolitan Asylums Board, various proposals for simplifying the whole machinery by which the accounts are rendered were agreed upon, and these have been embodied in the Bill. Part I. of the Bill seeks to legalise the position of local authorities who undertook to continue to make payments to officers and servants who joined His Majesty's Forces.

### Hospital Arrangements in Mesopotamia.

It is evident from the persistent questions asked in the House of Commons relative to the breakdown of the medical arrangements in Mesopotamia that members are determined that the whole unfortunate affair shall be thoroughly sifted, and the responsibility properly apportioned. Mr. R. Gwynne asked the Secretary for India to state to what extent Sir William Meyer was responsible for the transport of hospital equipment, and Mr. Chamberlain replied: "Sir W. Meyer is the Finance Member of the Government of India. He has no responsibility for the transport of hospital equipment for Mesopotamia, except such as may be held to attach to every member of the Government." Mr. Gwynne then asked who was responsible for the equipment and supply, and if those who had charge of these arrangements still occupied responsible positions. To this Mr. Chamberlain replied: "The Government of India are responsible for the equipment of all Forces coming from India, the War Office for those coming from Europe. Supply is arranged for by the Government of India in connection with the India Office or the War Office, according to the nature of the supply. The answer to the second question is in the affirmative." Replying to further questions, Mr. Chamberlain said that the inquiry which was being made regarding the medical and hospital arrangements of the Mesopotamian campaign would embrace all matters relative to the sufficiency or otherwise of the provision made in India and Mesopotamia for medical *personnel*, stores, and appliances; also as to the organisation of medical relief and the arrangements for dealing with the sick and wounded in the field, at the base, and in the transit to the base. He had issued instructions that the Commission should fearlessly ascertain and assign the responsibility for the breakdown of relief, whether individuals or the system were to blame. At the invitation of the Viceroy of India Mr. E. A. Ridsdale, Red Cross Commissioner, is joining the Commission of Inquiry as a member. Replying to further questions, Mr. Tennant said that all the resources at the disposal of the War Office, whether in *personnel* or stores, had been offered to the Commander-in-Chief in India, and that aid from Egypt was offered as soon as it was realised that assistance was wanted.

### Venereal Disease.

Various members have again urged the desirability of taking steps to frame a Bill with as little delay as possible, with the object of giving effect to the recommendations of the Royal Commission on Venereal Disease. It is understood that the President of the Local Government Board is considering whether a clause might be added to the Local Government (Emergency Provision) Bill, which would bring within its purview the recommendation that drugs used for the treatment of venereal disease should in certain cases be provided out of public funds, and that statistical information should be compiled as regards the prevalence of the disease among persons receiving institutional treatment.

### Other Matters of Interest.

Soldiers' Letters from Hospitals.—There are, according to a statement made by the Postmaster-General, serious objections, both in principle and practice, to exempting letters from soldiers in hospitals in this country from prepayment of postage.

"Degrading Initials."—The attention of the Under-Secretary for War was drawn to a newspaper article in which the words "degrading initials" were applied to the badges of the Royal Army Medical Corps and the Army Service Corps, but his view was that the article was written in a sarcastic spirit, and was not intended to cast ridicule on any branch of the Regular Forces.

Bone-setters and Wounded Soldiers.—Replying to Sir A. Markham, Mr. Tennant said the War Office had declined to accept the services of Mr. H. A. Barker, together with those of several other gentlemen who are not registered practitioners. He added that if the Army Council directed medical authorities to employ unqualified medical men to treat military patients, he felt quite certain that from no quarter would there be a louder outcry that our soldiers were not receiving skilful attention than from members of Parliament.

The Disabilities of Uninoculated Men.—Replying to Mr. Anderson, Mr. Tennant said the necessity of preventing the introduction of enteric fever into military camps was paramount, and this necessitated that leave should be granted sparingly to men who object to inoculation. He did not think the suggestion that if such men produced evidence from the medical officer of health for the district they intended to visit that the neighbourhood was free from typhoid fever provided sufficient safeguard that uninoculated men on leave would not bring back infection.

